# The functions of herpesvirus shuttling proteins in the virus lifecycle

**DOI:** 10.3389/fmicb.2025.1515241

**Published:** 2025-02-05

**Authors:** Huijun Cao, Mingshu Wang, Anchun Cheng, Bin Tian, Qiao Yang, Xumin Ou, Di Sun, Yu He, Zhen Wu, Xinxin Zhao, Ying Wu, Shaqiu Zhang, Juan Huang, YanLing Yu, Ling Zhang, Shun Chen, Mafeng Liu, Dekang Zhu, Renyong Jia

**Affiliations:** ^1^Engineering Research Center of Southwest Animal Disease Prevention and Control Technology, Ministry of Education of the People's Republic of China, Chengdu, China; ^2^Key Laboratory of Animal Disease and Human Health of Sichuan Province, Chengdu, China; ^3^International Joint Research Center for Animal Disease Prevention and Control of Sichuan Province, Chengdu, China; ^4^Institute of Veterinary Medicine and Immunology, Sichuan Agricultural University, Chengdu, China; ^5^Research Center of Avian Disease, College of Veterinary Medicine, Sichuan Agricultural University, Chengdu, China

**Keywords:** herpesvirus, shuttling proteins, nuclear localization signal, nuclear export signal, life cycle, innate immune escape, apoptosis

## Abstract

During viral infection, the transport of various proteins between the nucleus and cytoplasm plays an important role in the viral lifecycle. Shuttling proteins are key factors in the transmission of nucleocytoplasmic information within cells and usually contain nuclear localization signals and nuclear export signals to mediate correct positioning for themselves and other proteins. The nucleocytoplasmic transport process is carried out through the nuclear pore complex on the nuclear envelope and is mediated by specific protein carriers. The viral proteins that function through nucleocytoplasmic shuttling in herpesviruses have gradually been identified as research advances. This article provides an overview of how shuttling proteins utilize nucleocytoplasmic shuttling signals and nuclear transport receptors for nucleocytoplasmic transport, as well as discusses how herpesvirus shuttling proteins enhance the effective infection of viruses by affecting their lifecycle and participating in innate immunity, this review provides a reference for understanding the pathogenesis of herpesvirus infection and determining new antiviral strategies.

## 1 Introduction

**Figure 1:**
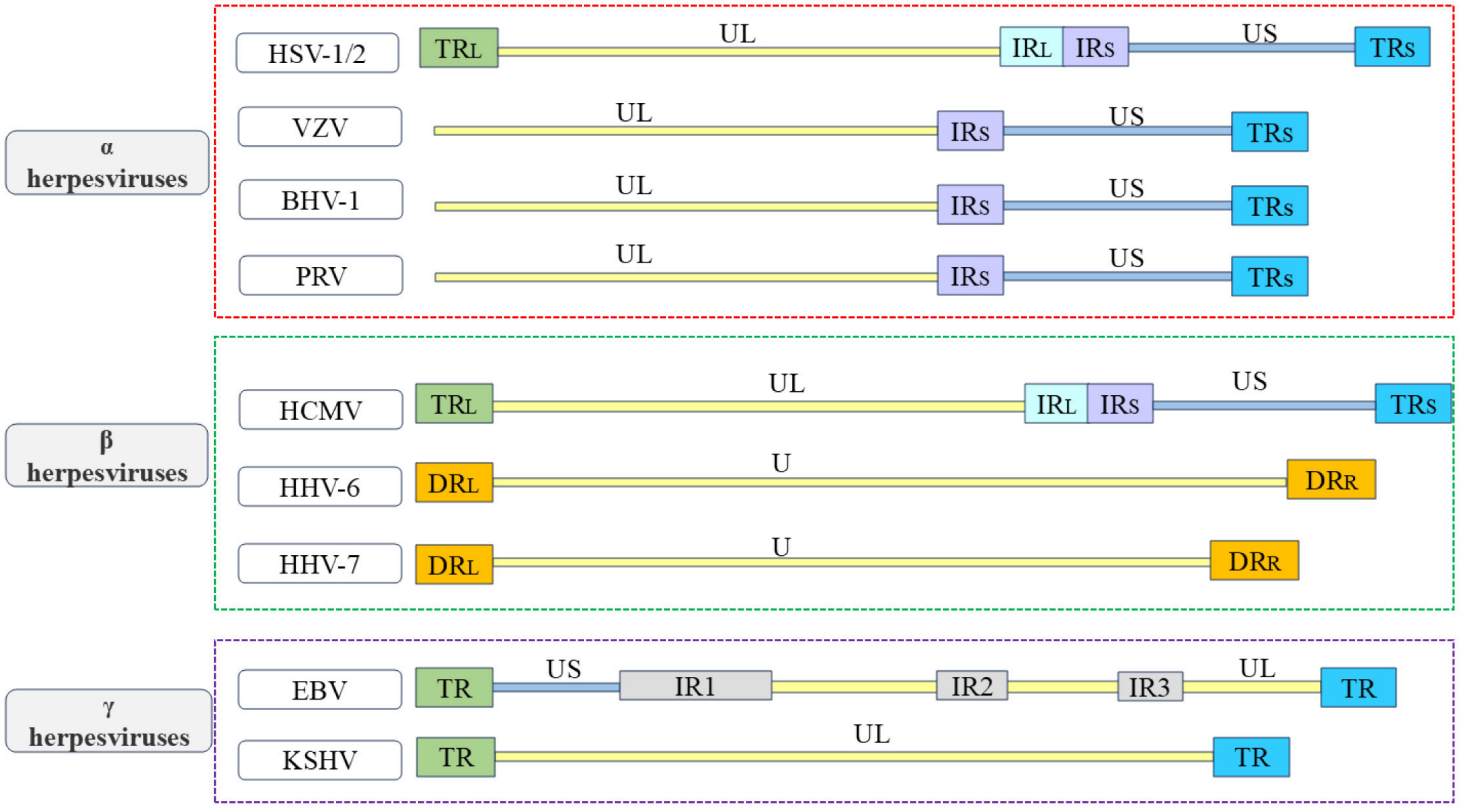
Overview of the structure of herpesvirus genome. In order to better understand the various types of herpesviruses and their associated viral proteins, the figure provides a brief overview of the genome structures of common α herpesviruses, β herpesviruses, and γ herpesviruses. The α herpesviruses include HSV-1, HSV-2, VZV, PRV, and BHV-1 (Davison and Scott, 1986; McGeoch et al., 1988, 1991; Klupp et al., 2005; Xu et al., 2015; Liu et al., 2022; Shitrit et al., 2023); the β herpesviruses include HCMV, HHV-6, and HHV-7 (Gompels et al., 1995; Dominguez et al., 1999; Dunn et al., 2003; Sijmons et al., 2014; Verbeek et al., 2024); the γ herpesviruses include EBV and KSHV (Russo et al., 1996; Kwok and Chiang, 2016; Majerciak et al., 2019). The repetitive sequences in the US are referred to as IR_S_ sequences and TR_S_ sequences. The repetitive sequences in the UL are referred to as IR_L_ sequences and TR_L_ sequences. The unique regions of the HHV-6 and HHV-7 genomes are collectively known as U.

Herpesviruses belong to the Herpesviridae family and are enveloped viruses. They have a large double-stranded nuclear genome. Herpesviruses can infect humans and other vertebrates, and employ a biphasic replication cycle consisting of latent and lytic phases, allowing the virus to infect the host and persist within it (Zhong et al., 1996; Davis et al., 2001; Fakhari and Dittmer, 2002; Klass et al., 2005; Fu and Pan, 2024). The herpesvirus family can be classified based on different physicochemical characteristics, such as cell tropism, pathogenicity, and latent sites, and can be divided into the α, β, and γ herpesvirus subfamilies (McGeoch et al., 1995; Boyne and Whitehouse, 2006a; Ilouze et al., 2006; Santos, 2016; Rathbun and Szpara, 2021). The α herpesvirus subfamily include herpes simplex virus type 1 and type 2 (HSV-1 and HSV-2), varicella zoster virus (VZV), which infect humans, as well as pseudorabies virus (PRV), Marek's disease virus (MDV), and bovine herpesvirus-1 (BHV-1), which infect animals. These viruses typically establish latency in sensory neurons (Biswas et al., 2013; Couteaudier and Denesvre, 2014; Depledge et al., 2018b; Suzich and Cliffe, 2018; Tognarelli et al., 2019; Zheng et al., 2022). The β herpesvirus subfamily, which include mainly human cytomegalovirus (HCMV), human herpesvirus 6 (HHV-6), and human herpesvirus 7 (HHV-7), which typically establish latency in mononuclear (Hahn et al., 1998; Agut et al., 2016; Elder and Sinclair, 2019; Hamada et al., 2023; Ijezie et al., 2023). The γ herpesvirus subfamily establishes a latent period in lymphocytes, including Kaposi's sarcoma-associated herpesvirus (KSHV) and Epstein-Barr virus (EBV), which infect humans; and alcelaphine herpesvirus 1 (AlHV-1) and herpesvirus saimiri (HVS), which infect animals (Russo et al., 1996; Dupin et al., 1999; Boshoff and Weiss, 2001; Bechtel et al., 2003; Thorley-Lawson et al., 2013; Myster et al., 2020; Damania et al., 2022). The herpesvirus particles are composed of a double-stranded DNA genome, capsid, tegument, and envelope from inside to outside, the herpesvirus genome contains multiple open reading frames (ORFs) that encode various viral proteins. The herpesvirus genome is covalently linked by a unique long region (UL) and a unique short region (US), with repetitive sequences at both ends of each unique region, including terminal repeat (TR) sequences, internal repeat (IR) sequences, and direct repeat (DR) sequences. Due to the variations in the location and quantity of repetitive sequences among different herpesviruses, the genome structures of the herpesviruses discussed in this review are briefly distinguished (see Figure 1; McGeoch et al., 1988, 1991; Russo et al., 1996; Rigoutsos et al., 2003). These viral proteins work separately and coordinate with each other to promote the proliferation and transmission of herpesvirus. The production of mature viral particles and the normal function of viral proteins are closely related to their correct subcellular location, viruses need to overcome multiple barriers, such as the plasma membrane and nuclear membrane, in host cells to enter the site of viral replication or assembly. Some viral proteins regulate the transcription and translation of host cells, innate immunity, and other biological functions by shuttling back and forth in the cytoplasm and nucleus and mediate viral mRNA transport, viral particle assembly, and interactions among various viral proteins to participate in the life cycle of the virus, thereby promoting the proliferation of the virus itself (Sandri-Goldin, 2001; Cullen, 2003; Lake and Hutt-Fletcher, 2004; Batisse et al., 2005; da Silva et al., 2012).

Throughout the lifecycle of herpesviruses, multiple viral proteins must function directly or indirectly at various locations. For example, the α herpesvirus HSV-1 UL47 protein and BHV-1 UL47 protein not only participate in the replication process as nuclear proteins but also play a role in primary envelopment during the maturation of viral particles (Donnelly et al., 2007; Liu et al., 2014b; Zhang et al., 2015, 2016). These viral proteins, which use specific sequences to dynamically change their localization and perform different functions, are called nucleocytoplasmic shuttling proteins, and their different localizations at different stages strongly impact their functions throughout the viral lifecycle. The specific shift sequences of shuttling proteins typically contain nuclear localization signals (NLSs) and nuclear export signals (NESs; Michael, 2000). In addition, a variety of receptors and adapter proteins are involved in the process of nucleocytoplasmic transport. Numerous herpesvirus shuttling proteins have been documented, classified according to α, β, and γ herpesviruses (Table 1). Among them, the ICP27 protein family is conserved in α, β, and γ herpesviruses, this family functions as a shuttling protein that regulates mRNA transport and influences the viral lifecycle (Patel et al., 2015; Tunnicliffe et al., 2015). A common feature of all herpesviruses is that most of the viral genes expressed in the lytic stage lack introns (Zheng, 2003). Since the herpesvirus gene is transcribed into mRNA in the nucleus of the host cell, it is necessary to export the intron-free viral mRNA to the cytoplasm for translation (Reed and Hurt, 2002; Le Hir et al., 2003). The ICP27 protein family translates viral mRNAs in the cytoplasm by hijacking the components of the host mRNA processing and export mechanism to ensure the expression and stability of herpesvirus intronless genes (Malik et al., 2004; Sandri-Goldin, 2008; Toth and Stamminger, 2008; Majerciak et al., 2014). In addition, the HSV-1 VP19C protein (Person and Desai, 1998; Adamson et al., 2006), HCMV UL94 protein, and KSHV ORF45 protein also affect the assembly of viral particles (Li and Zhu, 2009; Liu et al., 2009, 2012; Wang et al., 2015). Herpesviruses cause lifelong infection by evading the host immune system and establishing latent infection. Multiple viral proteins, including the PRV UL46 protein (Xu et al., 2020), HSV-1 γ_1_34.5 protein (Cheng et al., 2002; Verpooten et al., 2009; Pan et al., 2018), HCMV UL94 protein, and KSHV LANA2 protein, regulate the localization of specific signal molecules to inhibit or block signal transduction pathways (Seo et al., 2004; Zou et al., 2020). This strategy is crucial for viruses to evade immune responses. Based on this, shuttling proteins are important factors in dynamically coordinating nucleocytoplasmic life activities, and herpesvirus shuttling proteins balance NLSs and NESs in space and time to optimize the time and amplitude of viral gene expression. The NLS and NES of herpesvirus shuttling proteins and their transport mechanisms must be determined to analyze the process of viral proliferation of herpesvirus. This review summarizes the transport mechanism of shuttling proteins and discusses the roles of various shuttling proteins in the various stages of the virus lifecycle in herpesvirus.

**Table 1:** Shuttling proteins in herpesviruses.

**Subfamily**	**Protein**	**Herpesvirus**	**NLS amino acid sequence**	**Transport receptors**	**NES amino acid sequence**	**Transport receptors**	**References**
α Herpesvirus	ICP27	HSV-1	ARRPCSPECRHGGKVARLQPPPTKAQPA	Importin-α, importin β1	DMLIDLGLDLDL	TAP/NXF1	(Mears et al., 1995; Sandri-Goldin, 1998)
	UL47	HSV-1	EPPRRRREGPRARRRA	Importin βs			(Donnelly and Elliott, 2001)
	UL3	HSV-1	RKPRK	Importin-α, importin β1	IRKDLRLSL	CRM1	(Zheng et al., 2011)
	γ_1_34.5	HSV-1	RADRARFRRRVAEAEAVIGPCLGPEARAR	Importin-α, importin β1	LPPRLALRLR	CRM1	(Cheng et al., 2002)
	VP19C	HSV-1	PRGSGPRRAAST		LERLFGRLRI	CRM1	(Li et al., 2012b; Zhao and Zheng, 2012)
	US3	HSV-2				CRM1	(Finnen et al., 2010, 2011)
	UL47	BHV-1	RRPR, PRVRRPRP	Importin βs	LSAYLTLFVAL, RGPNHGAGDAMDTDAPPERAPEGGAPQD	CRM1	(Zheng et al., 2004; Verhagen et al., 2006)
	VP22	BHV-1	PRPR	Importin βs	LDRMLKSAAIRIL	CRM1	(Zheng et al., 2005)
	ICP27	BHV-1	RRAR	Importin βs	LEELCAARRLSL	CRM1	(Guo et al., 2009; Ding et al., 2010)
	IE4	VZV	RKHRDRSLSNRRRRP	Importin-α, importin β1		TAP/NXF1	(Huang et al., 2014)
	ORF10	VZV	RRR, KRK	Importin β1	LARLLYLHLYL	CRM1	(Cai et al., 2012)
	UL54	PRV	RQRRR	Importin-α, importin β1		TAP/NXF1	(Li et al., 2011a,b)
	UL46	PRV	RRARGTRRASWKDASR	Importin βs		CRM1	(Xu et al., 2020)
	ORF12	MDV	RSRSRSRSRERRRRRPRVRPGRR	Importin βs			(Schippers et al., 2015)
	UL54	DEV	KKKPSDHDTGKYVKRARA, PPNRDRRRMSDKSDFKQSRRSQR, RRVSWHTLCLIGKELRR	Importin-α, importin β1	LKLKLRPIFL		(Liu et al., 2016)
β Herpesvirus	UL94	HCMV	KLVGKSRKHR, RRRRR		CILCQLLLLY	CRM1	(Liu et al., 2012)
	UL84	HCMV	PEKKKEKQEKK	Importin-α, importin β1	LSLNLFALRI, LTLSSLTL	CRM1	(Xu et al., 2002; Lischka et al., 2006a)
	UL69	HCMV	ERRARRARRFCLDYEPVPRKFRRER	Importin-α, importin β1	APPAQPPSQPQQHYSEGELEEDEDSDDA		(Lischka et al., 2001; Toth et al., 2006)
γ Herpesvirus	BLLF2	EBV	KRQALETVPHPQNRGR, RRPRPPVAKRRRFPR	Importin β1		TAP/NXF1	(Li et al., 2021)
	EB2	EBV	KRRR, KRR	Importin βs		TAP/NXF1	(Hiriart et al., 2003b; Juillard et al., 2009)
	BFLF2	EBV	RRLMHPHHRNYTASKASAH			TAP/NXF1	(Li et al., 2018)
	LANA2	KSHV			MVPLVIKLRL	CRM1	(Muñoz-Fontela et al., 2003, 2005)
	ORF45	KSHV	KRKR	Importin β1	VLSQRIGLMDV	CRM1	(Li and Zhu, 2009)
	ORF57	KSHV				TAP/NXF1	(Bello et al., 1999)
	ORF57	HVS			ILPKSGEPKLFL	TAP/NXF1	(Goodwin et al., 1999)
	ORF57	AlHV-1					(Leenadevi and Dalziel, 2009)

## 2 The nucleocytoplasmic shuttling mechanism of shuttling proteins

Herpesvirus shuttling proteins have nucleocytoplasmic shuttling mechanisms similar to that of most other shuttling proteins. Each step is accompanied by many interactions between proteins and is subject to strict and complex regulation, as will be detailed below (Mattaj and Englmeier, 1998).

### 2.1 Channels for nucleocytoplasmic shuttling

The cytoplasm and nucleus are separated by nuclear envelope (NE), providing a physical barrier for the diffusion of large molecules between the cytoplasm and nucleus (Dey and Baum, 2021). NE is penetrated by multiple supramolecular structures, which are called nuclear pore complex (NPC), NPCs are complex structures composed of nucleoporins (Nups) that penetrate and bridge the inner and outer nuclear membranes, where they mediate nucleocytoplasmic transport (Strambio-De-Castillia et al., 2010; Knockenhauer and Schwartz, 2016; Fontana et al., 2022).

### 2.2 Transport receptors of nucleocytoplasmic shuttling

The nucleocytoplasmic shuttling of proteins around NPCs is mediated by specific protein carriers, collectively known as nuclear transport receptors (NTRs), such as karyopherins, which are responsible for transporting nucleocytoplasmic cargo (Bednenko et al., 2003; Mosammaparast and Pemberton, 2004; Conti et al., 2006). The functions of karyopherins can be divided into two different categories: importins and exportins, importins are heterodimers, which are divided into importin-αs and importin βs (Goldfarb et al., 2004; Xu et al., 2010). The importin-αs protein recognizes the NLS in the cargo protein for transport (Goldfarb et al., 2004). It has three key domains: its N-terminal domain is an IBB domain that binds importin β1, its middle domain is an Armadillo (ARM) repeat sequence that binds NLS-cargo, and its C-terminal region interacts with nuclear export factor CAS and nucleoporin 50 (Nup50) to mediate the nucleocytoplasmic transport of the protein (Görlich et al., 1996; Weis et al., 1996; Kutay et al., 1997; Herold et al., 1998).

**Figure 2:**
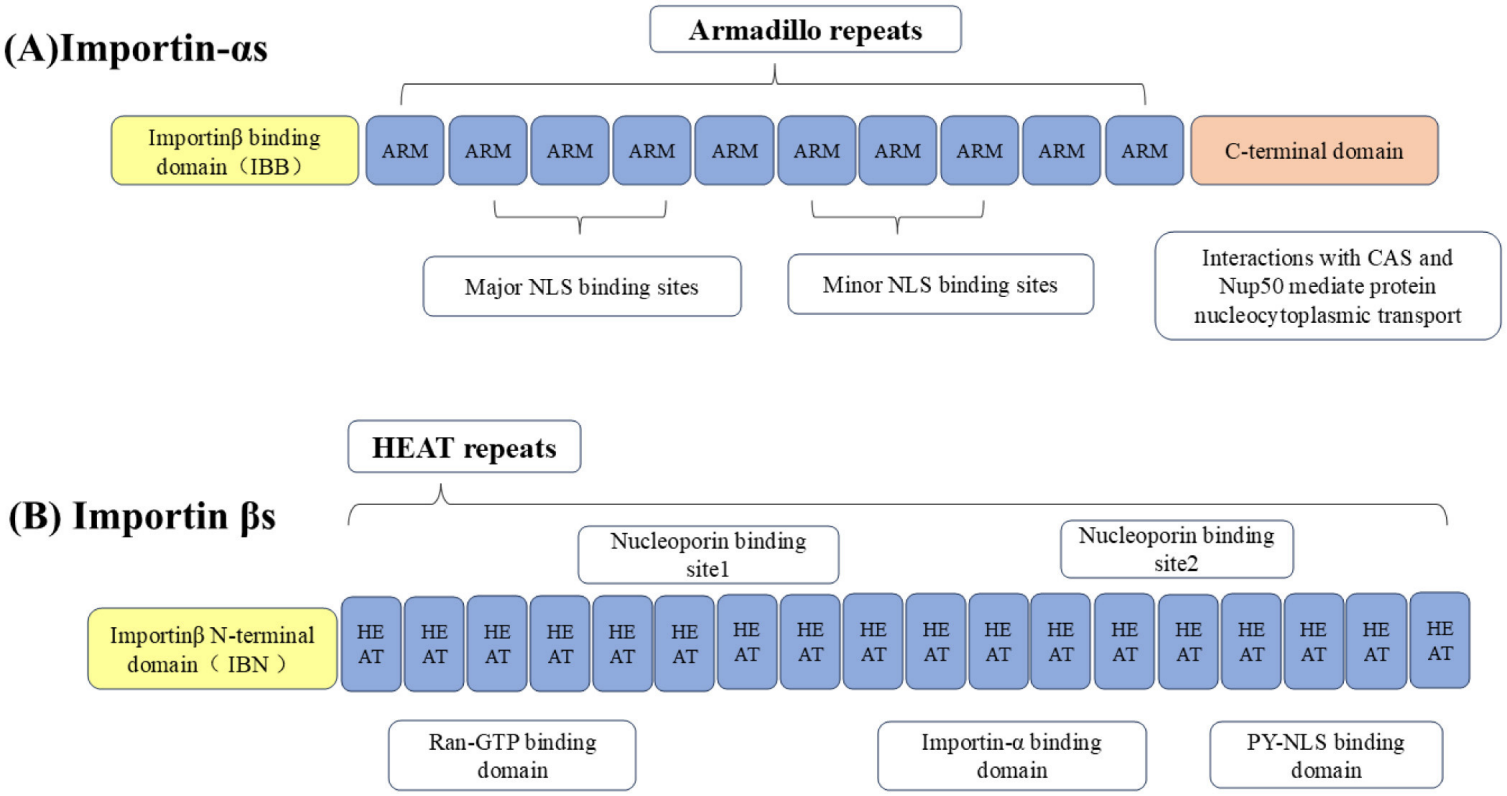
Importins are divided into **(A)** importin-αs and **(B)** importin βs. The N-terminus of importin-αs binds to the IBB domain of importin β1, the middle part is the Arm repeat sequence that bind to NLS-containing cargo, and the C-terminal region interacts with CAS and Nup50 to mediate nucleocytoplasmic transport of the protein. Importin βs contain two conserved domains, including the central HEAT domain and the importin β N-terminal domain (IBN).

Importin βs, another family of proteins involved in the nucleocytoplasmic transport of proteins and RNA. Almost all importin βs contain two conserved domains: the central HEAT domain and the importin β N-terminal domain (IBN), HEAT is named by the initials of the four proteins that initially found this repetitive motif (Figure 2; Xu et al., 2010). Importin βs include nuclear import receptors (importins), export receptors (exportins), and bidirectional receptors. For example, the human genome encodes 20 importin βs, of which 10 (importin β1, transportin 1, transportin 2, importin 4, importin 5, importin 7, importin 8, importin 9, importin 11, and importin 12) are importins, 7 (exportin 1/CRM1, exportin 2/CAS, exportin 5, exportin 6, exportin 7, exportin t, and RanBP17) are exportins, 2 (importin 13 and exportin 4) are bidirectional receptors, and 1 (RanBP6) has not been characterized (Nehrbass and Blobel, 1996; Kimura and Imamoto, 2014).

### 2.3 The energy sources for nucleocytoplasmic transport

The transport process of shuttling proteins to nuclear transport receptors depends on the participation of an active energy transport mechanism, which is generally driven by the RanGTP-RanGDP gradient during nuclear envelopment. Ran is the most abundant member of the Ras-GTPase superfamily and is a key regulator of the import protein importin β, which is crucial for protein and RNA transport between the nucleus and cytoplasm (Izaurralde et al., 1997; Görlich and Kutay, 1999). To sum up, NPC performs complex biological functions through the spatial and temporal interactions of cargo proteins, Ran GTPases, and NTR.

### 2.4 Nucleocytoplasmic shuttling of proteins into the nucleus

The NLS is a short peptide containing a special amino acid sequence that acts as a signal fragment that mediates the transport of shuttling proteins from the cytoplasm to the nucleus through the NPC, with “localization” and “orientation.” The NLS is divided into classical nuclear localization signals (cNLSs) and non-classical nuclear localization signals (ncNLSs; Table 2; Bradley et al., 2007). Unlike cNLSs (Robbins et al., 1991; Arregi et al., 2011). Many proteins contain NLSs with irregular characteristic structures, known as ncNLSs. There are many types of ncNLSs. These can generally be divided into NLSs rich in arginine (IK-NLSs), NLSs containing proline and tyrosine (PY-NLSs), and spatial epitope NLSs (Fagerlund et al., 2002; Meyer and Vinkemeier, 2004). The IK-NLS is recognized by importin5 (Kobayashi and Matsuura, 2013), while the PY-NLS is more complex than the cNLS and is recognized by transportin 1 (Lee et al., 2006; Wang et al., 2012; Mallet and Bachand, 2013; Soniat and Chook, 2016).

**Table 2:** Examples of different types of nuclear localization signal.

**NLS types**		**Consensus sequence**	**Typical sequence representative**	**References**
Classical nuclear localization signals (cNLS)	Monopartite type NLS (MP NLS)	K(K/R)X(K/R)	SV40 T antigen NLS(PKKKRKV)	(Kalderon et al., 1984)
	Bipartite type NLS (BP NLS)	R/K(X)10-12KRXK	Xenopus laevis nucleoplasmin NLS(KRPAATKKAGQAKKKK)	(Dingwall et al., 1988)
Non-classical nuclear localization signals (ncNLS)	IK-NLS	K(V/I)X-K-X 1-2(K/H/R)	Pho4 NLS (ANKVTKNKSN)	(Kaffman et al., 1998)
	PY-NLS	R/H/K-(X)2-5-PY	M9NLS (FGYNNQSSNFGPMKGGNFGGRSS GPY)	(Siomi and Dreyfuss, 1995)
Other types of nuclear localization	Spatial epitope NLS	/	STAT1	(Meyer and Vinkemeier, 2004)

In the classical nuclear import process, cNLS-cargo in the cytoplasm first binds to importin-α and then binds to importin β1 through the IBB domain form the cNLS-Cargo-importin-α-importin β1 trimer. Importin β1 then facilitates the transport of the trimer into the nucleus. The energy consumption of the trimer passing through the NPC is provided by the hydrolysis of GTP by Ran GTPase, and the combination of Ran GTP and importin β1 results in conformational changes in importin-α and cNLS-cargo, respectively, which are released from the trimer. cNLS-cargo is transported along the internal skeleton solid phase and remains in the nucleus (Lange et al., 2007; Stewart, 2007; Fontana et al., 2022). Non-classical nuclear entry is generally mediated by importin βs binding to the ncNLS, of which transportin 1 may be second only to importin-α1. The protein containing the PY-NLS appears to be imported specifically by transportin 1 (Siomi et al., 1997; Twyffels et al., 2014; Hwang et al., 2022). In summary, the process of NLS-mediated protein entry into the nucleus is a necessary factor for the proper functioning of the protein.

### 2.5 Nucleocytoplasmic shuttling of proteins out of the nucleus

**Figure 3:**
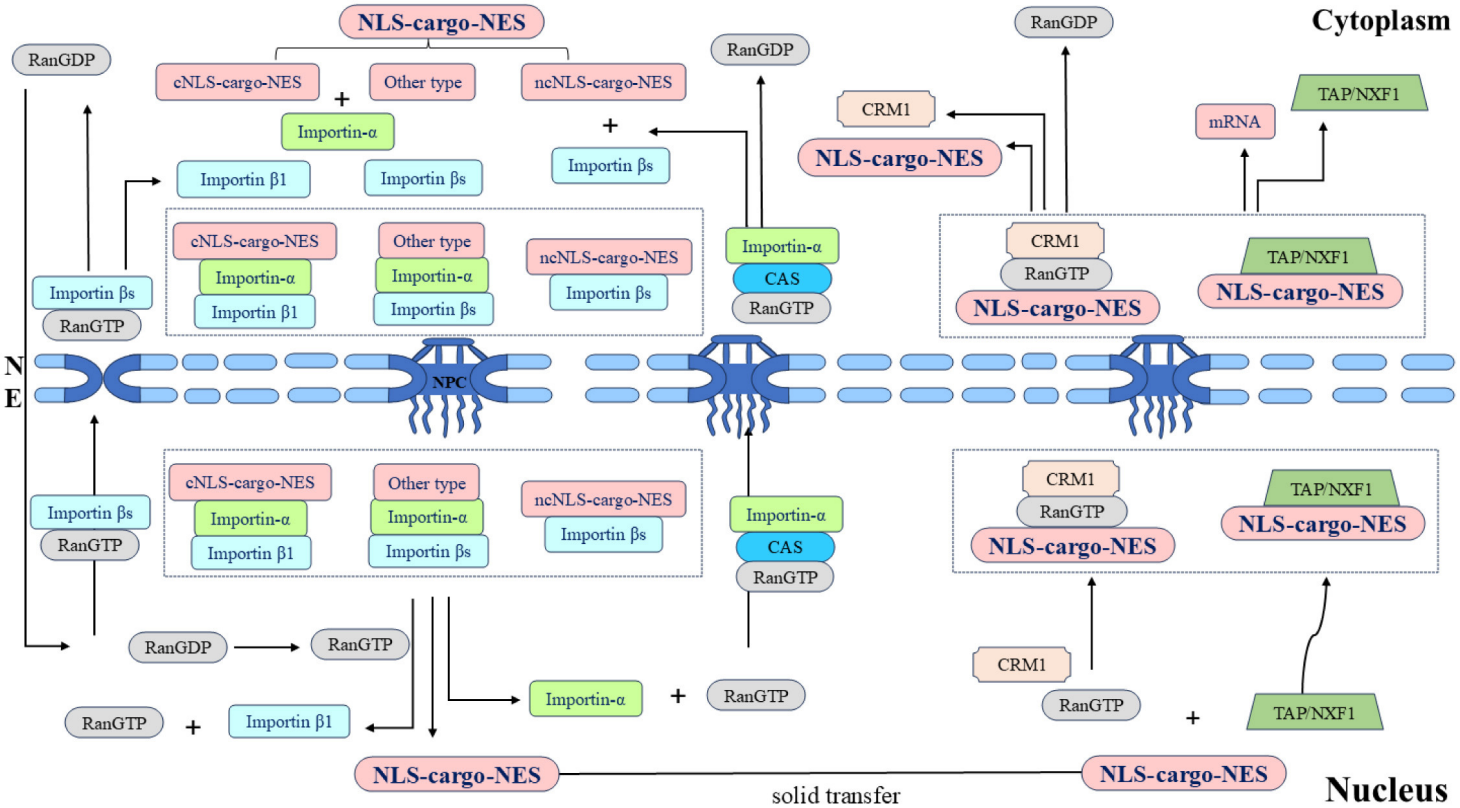
Schematic diagram of the nucleocytoplasmic transport of the shuttling protein NLS-cargo-NES protein complex and its associated protein molecular circulation. Different types of NLSs on shuttling proteins are recognized by different importins, and transport complexes are formed under the action of multiple factors. Importins directs the complex to the NPC and transfers it to the nucleus. Shuttling proteins undergo solid-phase transport within the nucleus, and different types of NESs on shuttling proteins are recognized by different exportins. The transport complex is formed under the coordination of various proteins, this complex is transported through the NPC to the cytoplasm, and the whole shuttling process is thus completed.

After the shuttling protein has completed its function in the nucleus, the process of gradual transport from the nucleus to the cytoplasm requires the NES to interact with exportins. The NES is a short, leucine-rich, or hydrophobic amino acid motif (Fischer et al., 1995; Wen et al., 1995). Among the seven types of exportins, CRM1, also known as export 1 or Xpo1, is the best characterized. The nuclear export process of shuttling proteins largely relies on CRM1 (Fornerod et al., 1997; Fukuda et al., 1997). First, the NES on the shuttling protein binds to CRM1 to form the CRM1-NES cargo RanGTP ternary export complex. The ternary complex then binds to various nuclear pore proteins and crosses the NPC to the cytoplasm. Finally, CRM1 returns to the nucleus through the NPC for the next export cycle (Kehlenbach et al., 1999; Seewald et al., 2002; Bernad et al., 2004; Hutten and Kehlenbach, 2006). In summary, the NES also plays an important role in shuttling protein transport, and the dynamic processes mediated by the NLS and NES are the basis for shuttling proteins to perform different functions (Figure 3).

## 3 The lifecycle of herpesviruses

### 3.1 Herpesvirus latency

When the virus is in the latency, the viral DNA is stably maintained in the nucleus of the cell as multiple copies of circular episomes, with the exception being the HHV-6, which has the ability to integrate into telomeric regions of host chromosomes (Arbuckle et al., 2010). In α herpesvirus, the latency-associated transcript (LAT) is the only viral gene expressed by HSV-1 (Whitley et al., 1998; Roizman et al., 2011; Wang et al., 2023). Infected cell protein 0 (ICP0) is an immediate-early regulatory protein of HSV-1 that promotes the lytic infection and reactivation of viral genomes (Leib et al., 1989; Halford and Schaffer, 2001; Thompson and Sawtell, 2006; Roizman et al., 2011). The expression of latent viral gene in VZV is also limited to VZV latency-associated transcript (VLT) and VLT-ORF63 RNA (VLT63) fusion transcripts (Depledge et al., 2018a; Ouwendijk et al., 2020; Braspenning et al., 2021). The tegument protein VZV IE62, encoded by the VZV gene, is delivered to the newly infected cell nucleus, where it initiates VZV lytic replication by transactivating viral immediate early, early, and late genes (Kinchington et al., 1992, 2001).

In β and γ herpesviruses, In HCMV, one of the mechanisms controlling the balance between latency and reactivation or lytic replication is the IE1 and IE2 proteins encoded by the HCMV UL123 and UL122, respectively. IE1 is essential for the establishment of lytic infection and the reactivation of viral latency (Tarrant-Elorza et al., 2014; Arend et al., 2016), IE2 initiates the subsequent cascade of viral gene expression (Malone et al., 1990; Dooley and O'Connor, 2020). Epstein-Barr nuclear antigen 1 (EBNA1) was the first reported EBV latency protein. The EBNA1 protein tethers the latent viral episomes to the host chromosome. According to the expression of viral genes, the latent infection of EBV is more complex and variable than those of other herpesviruses. The latent form of EBV infection is further categorized into five patterns: Latency 0, I, IIa, IIb, and III (McKenzie and El-Guindy, 2015; Thorley-Lawson, 2015). The lytic cycle activation of EBV is controlled by two viral transcription factors Zta and Rta, which are immediate early proteins encoded by EBV genes BRLF1 and BZLF1, respectively (Murata et al., 2021; Ali et al., 2022). The latency of KSHV also expresses only a limited number of viral genes, latency-associated nuclear antigen 1 (LANA1), encoded by the KSHV gene, is the main regulator of latency (Katano, 2018). The key viral protein that regulates the transition from latency to lysis replication is the transcriptional activator regulator (RTA), which is encoded by the KSHV UL50 gene (Miller et al., 2007; Srivastava et al., 2023).

To sum up, there are differences in the latency of herpesviruses. The α herpesviruses (such as HSV-1 and VZV) lurk in non-dividing neuronal cells. It is not necessary to express viral proteins to tether the viral genome to chromosomes. The β and γ herpesviruses (such as HCMV, EBV, and KSHV) that maintain latency in dividing cells express viral proteins, tethering the viral genome to chromosomes so that episomes are partitioned to daughter cells. Therefore, the viral proteins required for maintaining latency and facilitating reactivation are also distinct. Despite these differences, herpesviruses of the three subfamilies also have similar lifecycles. The differences and similarities among various herpesviruses during lytic replication are outlined below to provide a clear understanding of the effects of herpesvirus shuttling proteins on the viral lifecycle.

### 3.2 Lysis replication of herpesviruses

#### 3.2.1 Entry of herpesviruses

The entry of α, β, and γ herpesviruses into cells requires the coordinated interaction of multiple glycoproteins on the surface of viral particles, these glycoproteins were named with letters and prefix g (glycosylation). First, herpesviruses attach to host cells through a variety of viral glycoproteins and multiple binding receptors but do not trigger entry. Then, herpesviruses directly fuse with the host cell membrane through the viral envelope (or enter specific cells through endocytosis; Eisenberg et al., 2012; Sathiyamoorthy et al., 2017; Weed and Nicola, 2017). Although herpesviruses of different types (such as HSV-1, HCMV, and EBV) infect a variety of host cells, they share a common entry mechanism: glycoprotein gB and the heterodimer gH-gL. But different herpesvirus subfamilies use distinct viral glycoprotein combinations (gD, gp42, and gH-gL) to bind to various cellular receptors (Compton et al., 1992; Miller and Hutt-Fletcher, 1992; Gerna et al., 2004; Ryckman et al., 2006; Hutt-Fletcher, 2007).

#### 3.2.2 Dissociation of tegument proteins and transport of capsids

After the herpesviruses enter the host cell, the viral capsid, and some tegument proteins enter the cytoplasm, and some tegument proteins are quickly dissociated from the capsid. Some tegument proteins are still attached to the capsid after entering the cell and interact with microtubules to transport the capsid to the nucleus. An opening is formed at a single vertex of the capsid. Some virus-encoded capsid proteins bind to the nuclear pore proteins Nup214 and Nup358 on the surface of cytoplasmic NPC to ensure that the capsid opening is docked with the nuclear pore and stimulates DNA release. Because the tightly packed negatively charged genome inside the capsid causes internal pressure, the high internal pressure, dozens of times the atmospheric pressures, promotes ejection of the genome from the capsid, overcoming the NPC permeability barrier and releasing the viral DNA into the nucleus (Jovasevic et al., 2008; Copeland et al., 2009; Pasdeloup et al., 2009; Huffman et al., 2017; Brandariz-Nuñez et al., 2019; Dünn-Kittenplon et al., 2021). The α herpesviruses (such as HSV-1), some tegument proteins need to be dissociated from the viral particles to function. The outer tegument proteins are first dissociated, and then the inner tegument proteins are dissociated. The first dissociated outer tegument protein is VP16 (Wysocka and Herr, 2003; Bohannon et al., 2013). The second dissociated tegument protein is VP13/14, an outer tegument protein encoded by the UL47 gene, which is also a shuttling protein that will be highlighted below. Followed by VP22 protein (encoded by UL49 gene; Morrison et al., 1998), the tegument proteins dissociated in sequence are involved in viral replication, among which UL47 and UL49 genes are unique to α herpesvirus. However, the dissociation patterns of tegument proteins in VZV, the β herpesviruses, and γ herpesviruses have not been extensively studied.

#### 3.2.3 Transcription, replication, and capsid assembly of viral genomes

After the viral DNA is released into the nucleus, the linear viral DNA begins to cyclize and replicate through the rolling circle process to form concatemer (McVoy and Adler, 1994; Colletti et al., 2004; Gualtiero et al., 2013; Packard and Dembowski, 2021). When the viral genome completes transcription, translation, and DNA replication, the capsid proteins enter the nucleus to form the basic assembly unit of the capsid and assemble into the nucleocapsid in the nucleus (Heming et al., 2017). The viral genome is transcribed in a cascade manner in the nucleus. The viral genes are divided into the immediate early (IE), early (E), and late (L) genes according to the order of transcriptional regulation. The IE gene is first transcribed and expressed under the action of a transcription activator, and its transcription occurs before viral DNA replication. First, the post-transcriptional IE viral mRNA is exported from the nucleus and synthesizes the IE protein in the cytoplasm. Then, the IE protein re-enters the nucleus to activate the transcription of the E gene. Finally, the E protein initiates and guides the transcription and expression of the L gene (Honess and Roizman, 1974; Rixon et al., 1996; Gruffat et al., 2016; Dembowski and DeLuca, 2018). In general, the IE protein initiates and guides the transcription and expression of E gene and L gene through nuclear export and nuclear import pathways (Hiriart et al., 2003b; Donnelly et al., 2007; Liu et al., 2009, 2014b). The ICP27 protein family plays a crucial role in the transport of viral mRNA during the viral lifecycle, which will be discussed in detail below.

The capsid assembly mechanism is considered highly similar in herpesviruses (Lye et al., 2017). The structure and function of the main capsid proteins in the Herpesviridae are also highly conserved (see Table 3), the following is an example of HSV-1. The major capsid proteins encoded by HSV-1 include the VP5, VP23, VP19C, VP26, UL26, UL26.5, and UL6 proteins (Brown and Newcomb, 2011; Döhner et al., 2021; Villanueva-Valencia et al., 2021). After the capsid protein is encoded in the cytoplasm, it forms a subcomplex of VP5-UL26.5 and VP23-VP19C. The NLS dependent on the VP19C and VP5 proteins is coenters the nucleus, and the capsid is assembled in the nucleus (Newcomb et al., 1993; Booy et al., 1994; Newcomb et al., 2001; Beard et al., 2002). The terminase complex (UL15, UL28, and UL33 proteins) recognizes the cis-regulatory “pac” motif in viral DNA. The viral DNA is cut twice and cut into a unit-length genome (Beard et al., 2002). The viral DNA is released into the capsid by interacting with UL6 protein (Varmuza and Smiley, 1985; Beard et al., 2002). Among these, the HSV-1 VP19C protein is the shuttling protein that will be highlighted next.

**Table 3:** Conserved capsid-associated proteins encoded by different herpesviruses.

	α **Herpesviruses**		β **Herpesviruses**		γ **Herpesviruses**		**Function**	**References**
**Viruses**	**HSV-1**	**VZV**	**HCMV**	**HHV-6**	**KSHV**	**EBV**		
Capsid proteins	UL19/VP5	ORF40	UL86	U57	ORF25	BCLF1	Major capsid protein: forms hexons and pentons	(Nealon et al., 2001; Liu and Zhou, 2007; Nguyen et al., 2008; Henson et al., 2009; Lebrun et al., 2014; Ruhge et al., 2018; Zhang et al., 2019)
	UL18/VP23	ORF41	UL85	U56	ORF26	BDLF1	The two together constitute triplexes	
	UL38/VP19C	ORF20	UL46	U29	ORF62	BORF1		
	UL35/VP26	ORF23	UL48a	U32	ORF65	BFRF3	Located at the tip of hexons	
	UL26	ORF33	UL80	U53	ORF17	BVRF2	Generates mature forms of scaffolding proteins	
	UL26.5	ORF33.5	UL80.5	U53.5	ORF17.5	BdRF1	Scaffolding protein removed from capsid during DNA packaging	
	UL6	ORF54	UL104	U76	ORF43	BBRF1	Portal protein: complexed with terminase subunit	
DNA-packaging proteins	UL28	ORF30	UL56	U40	ORF7	BALF3	Terminase complex	(Visalli et al., 2015; Neuber et al., 2017; Visalli et al., 2019; Huet et al., 2020; Iwaisako et al., 2023; Iwaisako and Fujimuro, 2024)
	UL15	ORF42/45	UL89	U66	ORF29	BGRF1/BDRF1		
	UL33	ORF25	UL51	U35	ORF67.5	BFRF1A		
	UL32	ORF26	UL52	U36	ORF68	BFLF1		
	UL25	ORF34	UL77	U50	ORF19	BVRF1		
	UL17	ORF43	UL93	U64	ORF32	BGLF1		

#### 3.2.4 Nuclear egress, secondary envelopment and release

The assembled nucleocapsid interacts with the nuclear envelope, budding at the inner nuclear membrane (INM) to complete primary envelopment (Mettenleiter et al., 2013), and then forms a nucleocapsid virion structure wrapped by the primary envelope in the perinuclear space, called perinuclear virions (PEVSs), followed by the de-envelopment of virions at the outer nuclear membrane (ONM; Bigalke and Heldwein, 2016). Eventually, naked capsids are released into the cytosol (Sonntag et al., 2017; Lv et al., 2019). The nucleocapsids released into the cytoplasm by all herpesvirus subfamilies will bind to tegument proteins in an orderly manner, obtain the secondary envelope by budding into the trans-Golgi network, and form mature virus particles (Hogue, 2021; Roller and Johnson, 2021). The secondary envelopment of different herpesviruses generally occurs in the Golgi apparatus, early endosomes, or autophagosomes. The Golgi apparatus is widely considered to be the site of secondary envelope formation (Gershon et al., 1994; Zhu et al., 1995; Granzow et al., 1997; Hambleton et al., 2004; Wisner and Johnson, 2004; Sugimoto et al., 2008; Hogue et al., 2014). Subsequently, the mature virus particles are transported to the cell membrane by cytoplasmic vesicles, and the virus particles are released to the outside of the cell through exocytosis, and finally the transmission of the virus particles between the cells is completed. Herpesviruses express two conserved and essential nuclear egress regulatory proteins (see Table 4), The homologous BFLF2 protein of the UL31 protein in EBV is a shuttling protein that will be discussed later.

**Table 4:** NEC core protein encoded by different herpesviruses.

	α **Herpesviruses**		β **Herpesviruses**		γ **Herpesviruses**		**References**
**Viruses**	**HSV-1**	**VZV**	**HCMV**	**MCMV**	**EBV**	**KSHV**	
Core NEC protein	UL34	ORF24	UL50	M50	BFRF1	ORF67	(Granato et al., 2008; Desai et al., 2012; Milbradt et al., 2012; Leigh et al., 2015; Takeshima et al., 2019; Häge et al., 2020; Schweininger et al., 2022)
	UL31	ORF27	UL53	M53	BFLF2	ORF69	

## 4 The role of herpesvirus shuttling proteins in the viral lifecycle

**Figure 4:**
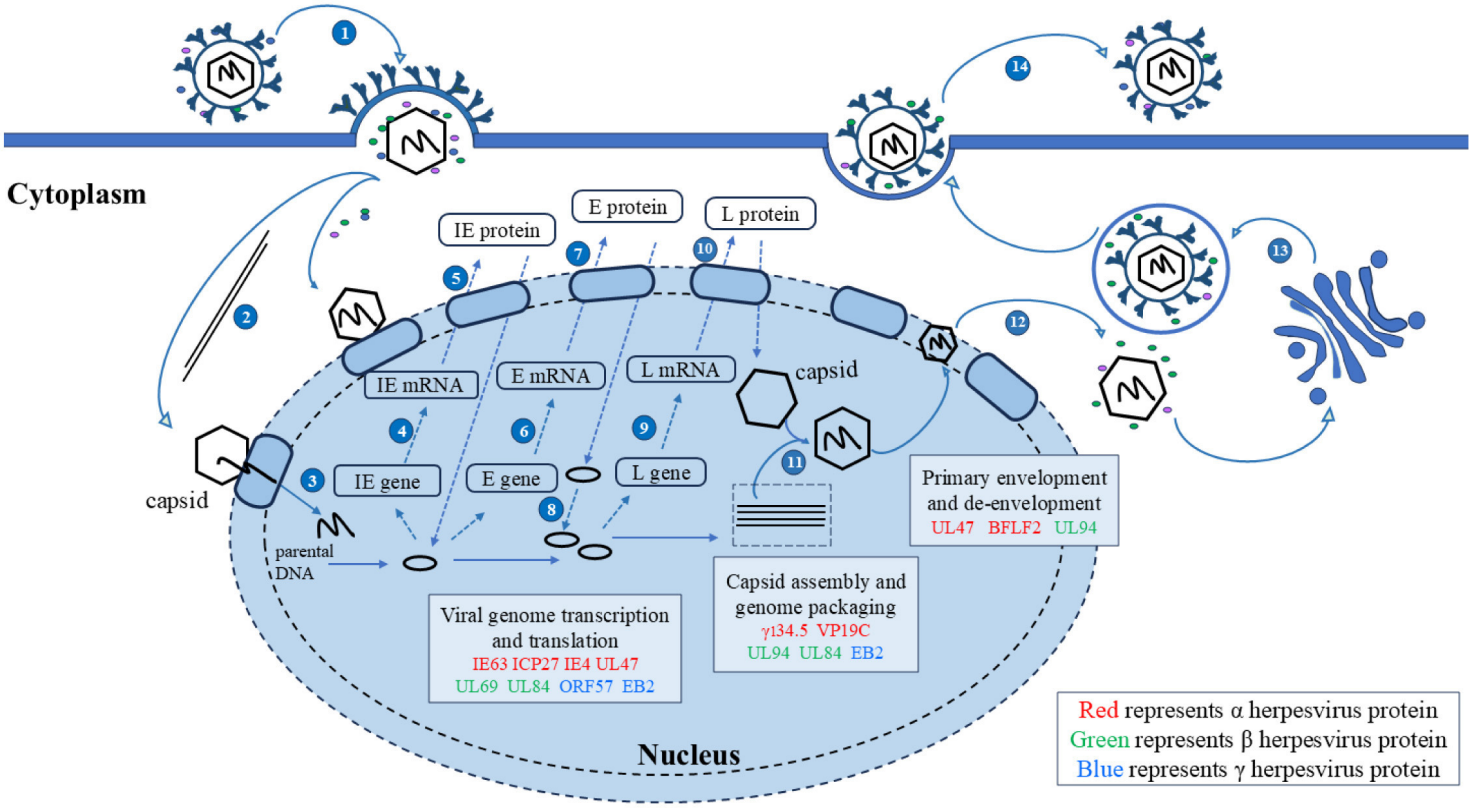
Herpesvirus shuttling proteins involved in the lifecycle of herpesviruses. 1. Entry; 2. The nucleocapsid enters the nucleus through microtubules; 3. Linear DNA is released into the nucleus; 4, 5. Transcription and translation of viral IE genes; 6, 7. Transcription and translation of viral E genes; 8. The viral genome replicates within the nucleus; 9, 10. Transcription and translation of viral IE genes; 11. Nucleocapsid formation and viral DNA packaging; 12. Primary envelopment and de-envelopment; 13. Secondary envelopment; 14. Release. Adapted from the literature (Sucharita et al., 2023; Li et al., 2024).

The mechanisms by which herpesviruses of different subfamilies infect host cells are slightly different. Herpesviruses have evolved different strategies to regulate the nucleocytoplasmic transport of NPC and proteins, creating an environment conducive to virus proliferation. Virus proteins target transport receptors through the NLS, NES, and other key functional domains, hijacking nucleocytoplasmic pathways to promote virus proliferation. Virus lifecycles depend on the transcription and replication in the nucleus of the host cell (Knipe, 1989; Boehmer and Lehman, 1997), and newly formed virus particles assemble into capsids in the nucleus (Nii et al., 1998). Herpesviruses must target transcription factors, scaffold proteins, and capsid proteins to the nucleus to participate in viral transcription, genome replication, capsid assembly (Malik et al., 1996; Rixon et al., 1996). The newly assembled viral particles from the capsid must subsequently leave the nucleus and continue to mature in the cytoplasm using tegument proteins and glycoproteins, the NES plays an important role in guiding the export of viral proteins into the cytoplasm. In summary, the nucleocytoplasmic shuttling proteins of herpesvirus ensure the correct cellular compartmentalization of herpesvirus proteins through interactions between the NLS and NES and transport receptors, which play important roles in the herpesvirus lifecycle. Mutations in NLS and NES affect the efficiency of nucleocytoplasmic transport to varying degrees. A detailed discussion of how herpesvirus shuttling proteins participate in processes throughout the lifecycle and what functions they play will be presented later (Figure 4).

### 4.1 Herpesvirus shuttling proteins affect viral replication

#### 4.1.1 Herpesvirus shuttling proteins regulate mRNA transport

**Figure 5:**
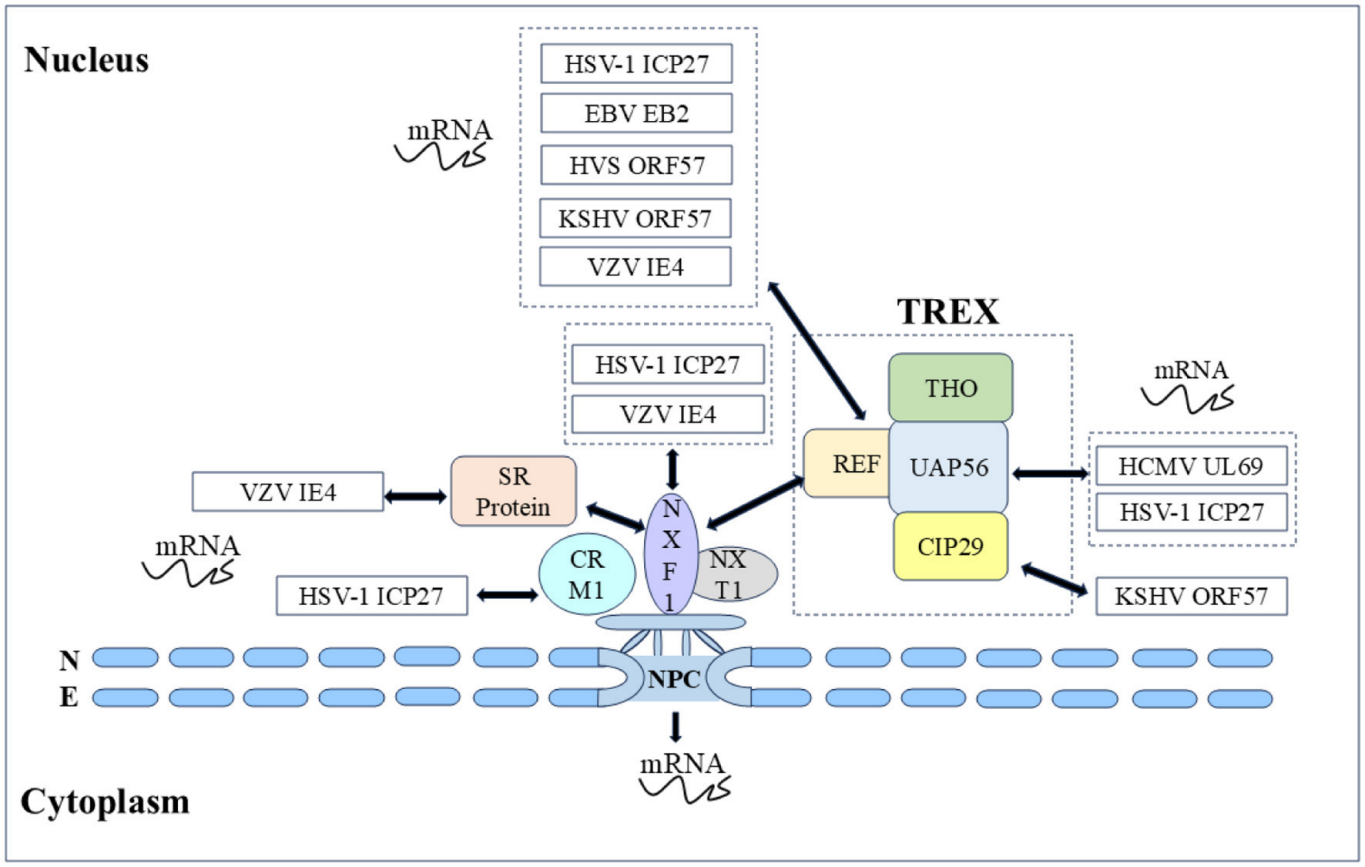
Herpesvirus shuttling proteins export mRNA by hijacking host mRNA export mechanisms (NXF1-mediated and CRM1-mediated). Double-headed arrow represents interaction.

mRNA transport is closely related to transcription, splicing, post-transcriptional modification, and translation, these processes are not isolated from each other but are highly continuous, dynamic, and complex. Most herpesvirus genes have no introns, and their replication depends on the selective nuclear export of intronless viral mRNAs. Since intronless virus mRNA cannot recruit mRNA export factors through a splicing-dependent mechanism, regulating nuclear mRNA export is crucial for the replication and pathogenesis of the herpesvirus (Gales et al., 2020). Research has shown that herpesvirus uses Aly/REF, TAP/NXF1, CRM1, and other export pathways to alter cellular and viral mRNA export and utilizes some regulatory proteins to promote viral mRNA nuclear export (Fornerod et al., 1997; Stade et al., 1997; Grüter et al., 1998), such as the ICP27 protein family encoded by herpesvirus, which includes the HSV-1 ICP27 protein (Johnson et al., 2009; Johnson and Sandri-Goldin, 2009; Koffa et al., 2023), VZV IE4 protein (Ote et al., 2009), BHV-1 ICP27 (Guo et al., 2009; Ding et al., 2010), HCMV UL69 protein (Zielke et al., 2011), EBV EB2 protein (Hiriart et al., 2003a), HVS ORF57 protein (Williams et al., 2005), and KSHV ORF57 protein (Boyne et al., 2008). They act as viral mRNA export factors, mediating the nucleocytoplasmic transport of viral transcripts (Figure 5).

In α herpesvirus, the HSV-1 ICP27 protein shuttle is coupled with viral mRNA export to the cytoplasm, and the nucleocytoplasmic shuttle activity depends on the NLS and NES as well as the other regions. Many studies have shown that the ICP27 protein recruits export factors such as Aly/REF, UAP56, and TAP to viral mRNA and promotes mRNA export through the TAP/NXF1 pathway. The ICP27 protein has been shown to affect the nucleocytoplasmic transport of UL15, UL17, and UL48 mRNAs, as well as the cytoplasmic levels of UL30, UL29, UL42, and UL5 mRNAs (Phelan et al., 1996; Uprichard and Knipe, 1996; Soliman et al., 1997). Initially, to screen the functional regions of the ICP27 protein, some scholars constructed 16 HSV-1 ICP27 mutants and finally reported that the mutations M11, M15, and M16 in these three regions completely inactivated the basic functions of the ICP27 protein (M11, M15, and M16 mutations led to amino acid substitutions at positions 340/341, 465/466, and 488, respectively). The shuttle ability of five HSV-1 ICP27 mutant proteins was subsequently studied, it was found that the M15 mutation affects the interaction between ICP27 and the core nucleoporin Nup62, causing M15 to be unable to re-export and shuttle continuously. After the M15 mutation completely blocks the shuttling activity of ICP27, it is not able to induce some viral late mRNAs, and seems owing to the loss of this key function, M15 mutant viruses are replication defective. In addition, the ICP27 NES mutation (Dleu) was found to weaken the shuttle, and Dleu mutant viruses were also replication defective (Rice and Lam, 1994; Mears and Rice, 1998; Lengyel et al., 2002; Malik et al., 2012). Subsequently, it was further found that the M15 mutant significantly reduced the interaction between ICP27 and mRNA, which explained the close relationship between the nucleocytoplasmic shuttling of ICP27 protein and mRNA (Sokolowski et al., 2003; Srinivas et al., 2021). Notably, the nucleocytoplasmic shuttling activity of the HSV-1 ICP27 protein is related to the activation of the late gene of the virus. How the shuttle of ICP27 facilitates the transcriptional activation of the late gene of the virus and whether ICP27 stimulates the nuclear export of the viral gene transcript by binding to the viral gene transcript in the nucleus and escorting it to the cytoplasm still need to be explored (Mears and Rice, 1998). The VZV IE4 protein possesses transactivating properties, and the IE4 protein can recruit the TAP/NXF1 receptor into viral mRNA to mediate mRNA export by interacting with the cellular export adapter protein SR protein. Some scholars have demonstrated that three domains of the IE4 protein, Ra, Rb, and Rc, mediate the interaction between the IE4 protein and its mRNA, Rb, and Rc not only participate in the transactivating properties of the IE4 protein but also mediate interactions with transcription factors such as p50 and TFIIB. Since the IE4 NLS is located in the Rb domain, the IE4 protein loses the ability to migrate to the nucleus when binding to mRNA and is exported to the cytoplasm, it is reasonable to speculate that only the IE4 NLS can localize to the nucleus to promote interaction with transcription factors (Baudoux et al., 2000; Ote et al., 2009, 2010; Huang et al., 2014). In addition, in the α herpesvirus UL47 protein family, the nucleocytoplasmic shuttling behaviors of the HSV-1 UL47 protein and BHV-1 UL47 protein are similar and are mediated by importin β for nuclear import (McLean et al., 1990; Donnelly and Elliott, 2001; Zheng et al., 2004), HSV-1 UL47 serves as an effective RNA-binding protein and its NLS and RNA binding sequence are NLS determined by the same motif. After binding to mRNA *in vivo*, the HSV-1 UL47 protein targets the main nuclear domain where viral mRNA transcription occurs (Donnelly et al., 2007).

In β herpesvirus, HCMV UL69 protein, as a post-transcriptional transactivator, promotes the nuclear export of mRNA through nucleocytoplasmic shuttling and the ability to recruit components of the cell mRNA export mechanism. UAP56 and URH49 are closely related RNA helicases that function in selective mRNA processing and export pathways, subsequent testing of the mRNA export activity of the HCMV UL69 protein revealed that the NLS and RNA binding motifs partially overlap with the UAP56/URH49 interaction motif, which is crucial for mRNA export activity, and that UAP56 binding-deficient viruses exhibit strong replication defects. In addition, the UL69 NES mutant, whose nucleocytoplasmic shuttling ability was lost, also lost the ability to activate gene expression and export mRNA (Lischka et al., 2001, 2006b; Stamminger, 2008; Zielke et al., 2011). Other UL69 homologous proteins (chimpanzee cytomegalovirus C69 protein, rhesus cytomegalovirus Rh69 protein, human herpesvirus 6 U42 protein, and elephant endotheliotropic herpesvirus U42 protein) in β herpesviruses were subsequently studied. Nucleocytoplasmic shuttling and homodimerization are conserved features of the HCMV UL69 homologous protein family of herpesviruses, whereas heterodimerization and recruitment of the cell mRNA export factors UAP56 and URH49 are limited to members of the UL69 protein (C69 protein and Rh69 protein) of the cytomegalovirus genus. All of these proteins function as viral mRNA export factors. In addition, HCMV UL84 protein is also a shuttling protein in HCMV that affects mRNA export, HCMV UL84 protein enhances the accumulation of viral transcripts encoding replication proteins in the cytoplasm. The UL84 protein enters the nucleus through interactions between unconventional nuclear-targeting domains and importin-α and participates in virus replication compartments by interacting with HCMV UL44 protein (polymerase accessory protein), which plays a direct role in DNA replication (Xu et al., 2002; Lischka et al., 2006a; Gao et al., 2010; Strang et al., 2012). UL84 is also an RNA binding protein, the construction of a non-shuttle mutant of UL84 NES revealed that the accumulation of HCMV IRS1 mRNA in the cytoplasm was reduced. The IRS1 protein is an immediate early protein encoded by the IRS1 ORF in the HCMV inverted repeat sequence. The IRS1 protein is involved in blocking the α group phosphorylation of eukaryotic initiation factor 2 (eIF2α) and the shutdown of cellular protein synthesis, and eIF2 is a key translation regulator. These results indicate that UL84 protein nucleocytoplasmic shuttling mediates IRS1 mRNA export and plays an important role in viral replication (Hakki et al., 2006; Gao et al., 2010). Members of the DExD box protein family are involved in various aspects of RNA metabolism, with many DExD/H proteins shuttling RNA from the nucleus to the cytoplasm. UL84 is a putative member of the DExD/H box protein family, further enhancing our understanding of UL84-mediated mRNA processes (Colletti et al., 2005).

In γ herpesvirus, the EBV EB2 protein has a similar ability to export mRNA, and the EB2 DN region binds to the export factor REF, which is critical for mRNA export (Hiriart et al., 2003b; Juillard et al., 2009). The nucleolus is the center of ribosome biogenesis and is involved in viral replication, and nucleolar localization sequence (NoLS) can target viral proteins to the nucleolus. The HVS ORF57 protein binds to viral mRNA for nucleocytoplasmic shuttling, and the nucleolar localization of ORF57 is crucial for nuclear mRNA export and gene activation. It has been reported that the ORF57 NLS mutants cannot be transport to the nucleolus reportedly, resulting in the loss of viral mRNA nuclear export, explaining the direct functional role of the nucleolar transport of viral proteins in viral mRNA nuclear export (Boyne and Whitehouse, 2006b). Notably, BHV-1 ICP27 NLS or NoLS single deletion mutants did not eliminate the transactivation activity of the glycoprotein gC promoter, whereas NLS and NoLS double deletion mutants lost this function. These results suggest that the nuclear and nucleolar localization of BHV-1 ICP27 may be involved in the regulation of viral RNA transport (Guo et al., 2009; Ding et al., 2010). The KSHV ORF57 protein interacts with the REF to recruit the transcription-export (TREX) complex to intron-free viral mRNA, enabling efficient mRNA export. During mRNA export, the ORF57 protein shuttles through the nucleolus, and the TREX complex is located in the nucleolus via the ORF57 protein. However, the exact cause of nucleolar localization during shuttling remains to be elucidated (Boyne et al., 2008; Boyne and Whitehouse, 2009; Jackson et al., 2011; Li et al., 2012a). Given the above, the ICP27 family, as posttranscriptional transactivators, promotes the nuclear export of mRNAs through nucleocytoplasmic shuttling and the ability to recruit components of the cellular mRNA export mechanism, strongly demonstrating the importance of nucleocytoplasmic shuttling in mediating the function of the ICP27 protein family.

#### 4.1.2 Herpesvirus shuttling proteins regulate mRNA stability

mRNA is easily destroyed by physical, chemical, and enzymatic factors, and its stability in cells is called mRNA stability, which is one of the basic mechanisms regulating gene expression and determines the final mRNA concentration at the post-transcriptional level (Liu et al., 2014a; Radhakrishnan and Green, 2016; Li et al., 2022; Watson and Thoreen, 2022). VHS is an endoribonuclease that is important for viral replication. VHS degrades cells and viral mRNA, the degradation of cellular mRNA to increase the availability of cellular translation mechanisms to promote the synthesis of viral proteins, viral mRNA degradation helps regulate the sequence expression of different viral genes (Taddeo and Roizman, 2006; Taddeo et al., 2006). In α herpesvirus, the HSV-1 UL47 protein interacts with VHS, assisting in the translocation of some VHS to the nucleus. In transfected cells, the VHS NES mutant can degrade stable mRNA, but it does not degrade in infected cells, suggesting that UL47 protein greatly weakens the degradation of viral mRNA (Shu et al., 2013a,b). In addition, KSHV ORF59 is a viral DNA polymerase processivity factor, and the KSHV ORF57 protein has been reported that interacts with the RNA export cofactors RBM15 and OTT3 through the NLS2 and NLS3 region to offset the nuclear accumulation of KSHV ORF59 mRNA, thereby promoting the expression of intronless ORF59 genes. RNA decay analyses after actinomycin D-mediated suppression of polymerase II transcription revealed that the ORF57 protein enhances the stability of ORF59 mRNA by increasing its half-life in cells (Nekorchuk et al., 2007; Majerciak et al., 2011). Overall, the ICP27 protein family plays a role in post-transcription, promoting mRNA nuclear export into the cytoplasm and acting as a posttranscriptional transactivator. However, the main function of the KSHV ORF57 protein is to stabilize mRNA. The HSV-1 UL47 protein regulates the cascade activation mechanism of viral genes and maintains the stability of viral mRNA by mediating the localization of intracellular VHS.

### 4.2 Herpesvirus shuttling protein affects virus particle assembly

The maturation of virions requires capsid assembly, primary envelopment, secondary envelopment, and other processes. Owing to the large nucleocapsid of herpesvirus, it passes through the INM and ONM through the nuclear pore. Therefore, herpesviruses have evolved a unique nuclear export mechanism. After successful assembly of the nucleocapsid, the offspring virions enter the perinuclear space between the INM and ONM for primary envelopment through budding, after which the nucleocapsid of the envelope fuses with the ONM, releasing the de-enveloped nucleocapsid into the cytosol. The unique nuclear export mechanism of viral particles allows tegument proteins to attach directly or indirectly to the nucleocapsid and exert their functions, facilitating the expulsion and maturation of viral particles from the nucleus (Mettenleiter et al., 2013; Owen et al., 2015).

In α herpesvirus, the HSV-1 UL47 protein and BHV-1 UL47 protein are the main components of the viral particle tegument and not only affect replication but also participate in virus assembly. Phosphorylated BHV-1 UL47 protein affects its cellular localization plays a role in viral DNA encapsidation and secondary virion incorporation (Zhang et al., 2015, 2016). The HSV-1 UL47 ineffective virus affects the nuclear export of virus particles, resulting in a decrease in the proportion of primary enveloped virus particles in the perinuclear space. According to reports, the accumulation of nuclear capsids and the lack of primary enveloped virions in the perinuclear space in the absence of the UL47 protein likely reflect an imbalance between the rate of virion delivery into the perinuclear space and the rate of egress from this region. The UL47 protein appears to be required for efficient primary envelopment of nucleocapsids in HSV-1 nuclear export, the cytoplasmic shuttle characteristic of the UL47 protein may be the cause of this phenomenon (Liu et al., 2014b). In addition, the HSV-1 UL47 protein interacts with the UL31/UL34 complex and US3, regulating their functions to promote the primary envelope of viral particles (Liu et al., 2014b). The HSV-1 γ_1_34.5 protein facilitates nuclear egress, and the absence of the γ_1_34.5 protein leads to the accumulation of nucleocapsids, further research revealed that deleting the amino-terminal nuclear egress domain (including nucleolar localization signals) of this protein increases the accumulation of capsid in the nucleus, indicating the importance of this domain for the function of the γ_1_34.5 protein, it is possible that this cis-element is required to direct virus egress from the nucleus to the cytoplasm (Brown et al., 1994; Cheng et al., 2002; Mao and Rosenthal, 2002; Jing et al., 2004). The HSV-1 VP19C protein is a structural protein of HSV-1 viral particles and is crucial for the assembly of the capsid. The VP19C protein utilizes NLS to bind to importin βs to cause nuclear import, and NES binds to CRM1 to cause export, becoming the first herpesvirus capsid protein with nucleocytoplasmic shuttling properties. The VP19C protein mediates its involvement in the assembly of the capsid structure, which can non-specifically bind to viral DNA and may play a role in anchoring DNA to the capsid in the nucleus (Braun et al., 1984; Tatman et al., 1994; Person and Desai, 1998). Another important function of the VP19C protein is to correctly transport component proteins to the capsid assembly site, where they serve as carriers to transport cytoplasmic capsid proteins (such as the VP23 and VP5 proteins) to the nucleus and promote capsid assembly. The nucleocytoplasmic shuttling ability of the VP19C protein is the direct condition for its function, and its NLS and NES mutations in recombinant viruses have demonstrated that the nuclear import of the VP19C protein is necessary for efficient production of HSV-1 (Rixon et al., 1996; Adamson et al., 2006; Okoye et al., 2006).

In β herpesvirus, the pp28 protein is a tegument protein encoded by HCMV UL99, HCMV pp28 mediates the capsid envelopment of HCMV and is an essential tegument protein required for HCMV assembly (Jones and Lee, 2004; Seo and Britt, 2006). The HCMV UL94 protein has been shown to interact with pp28 in the virus assembly compartment (AC), and a mutation at 343 aa has been found to alter the cytoplasmic distribution of the UL94 protein and disrupt its nucleocytoplasmic shuttling. The 343 aa site of the UL94 protein is the nuclear import site and a key site for interaction with pp28, which can serve as a bridge connecting the capsid and envelope. The UL94 protein partially guides pp28 to the assembly complex and participates in the secondary envelope of viral particles, demonstrating the importance of UL94's nucleocytoplasmic shuttling properties in mediating pp28's participation in the assembly process of the virus (Liu et al., 2009, 2012).

The shuttling proteins affecting viral assembly in γ herpesviruses include the KSHV ORF45 protein, the EBV EB2 protein, and the EBV BFLF2 protein. The KSHV ORF45 protein is both a tegument protein and an immediate early protein that is necessary for the entry and release of the targeted viral capsid. The viral particle protein content was significantly reduced in the BAC-45rc mutant in which the ORF45 NLS was disrupted (Li and Zhu, 2009). The lysine in the NLS often serves as a ubiquitin acceptor site (Chan et al., 2006), Lys297 in the ORF45 NLS is critical for ORF45 targeting of lipid rafts (LRs) and subsequently guides viral particles to the Golgi and endosome membrane for budding (Wang et al., 2015). In BCBL-1 cells, a portion of the ORF45 protein was found to be colocalized with the viral replication compartment, suggesting that the binding of the ORF45 protein to the capsid may participate in the maturation processes of the virus. However, further studies are needed to clarify the role of the ORF45 protein in the late stages of viral replication (Kuang et al., 2008; Li and Zhu, 2009). EB2 is a nuclear protein that mediates mRNA nuclear export. Studies have shown that in the absence of EB2, the newly replicated viral DNA is not properly encapsidated and is eventually completely digested by DNase I, suggesting that the correct balance of protein expression involved in intranuclear capsid assembly and maturation can be obtained only in the presence of EB2. In addition, as EB2 is crucial for the nuclear export of most late mRNAs and these genes are converted into proteins involved in nuclear capsid assembly and maturation, EB2 is essential for the correct assembly of nuclear capsids (Batisse et al., 2005). The EBV BFLF2 protein belongs to the herpesvirus UL31 protein family, and the EBV BFRF1 protein belongs to the herpesvirus UL34 protein family. The UL31 protein and the UL34 protein family are conserved among all herpesviruses and are the main viral components involved in the primary envelope of the virus, the UL31 protein and the UL34 protein family form a nuclear export complex to mediate the nuclear export of herpesvirus. BFLF2 is associated with the nuclear export of TAP and interacts with importin-α7, importin β1, and transportin 1, promoting the entry of the EBV nucleocapsid from the nucleus into the cytoplasm for the subsequent maturation of viral particles. Transfection of BFLF2 and BFRF1 interacting region deletion mutants, as well as NLS mutants, resulted in a significant decrease in the level of secreted viral particles. Both the nuclear targeting of BFLF2 and the BFRF1-interacting domains of BFLF2 are necessary for EBV maturation and secretion (Gonnella et al., 2005; Li et al., 2018; Dai et al., 2020). To sum up, nucleocytoplasmic shuttling proteins in herpesviruses affect the assembly of viral particles by participating in nucleocapsid assembly and influencing the primary envelopment of the virus.

### 4.3 Interactions of herpesvirus shuttling proteins with other proteins affect viral replication

Herpesvirus shuttling proteins interact with other proteins and regulate orderly in different compartments. In α herpesvirus, the UL47 protein forms a stable complex with the US3 protein in HSV-1 infected cells, and the UL47 protein is required for effective nuclear localization of the US3 protein. HSV-1 US3 plays an important role in the viral lifecycle by phosphorylating a variety of viral and host proteins, such as regulating cell morphology or microtubule networks and promoting the nuclear egress of progeny nucleocapsids. A study showed that phosphorylation of UL47 Ser77 (adjacent to the UL47 NLS) by US3 regulates the nuclear localization of the UL47 protein, and mutations in this site affect the effective viral replication of HSV-1 in mice (Kato et al., 2011). Further study demonstrated that the UL47 protein, UL34/UL31 complex and US3 protein colocalize to the nuclear envelope and promote viral nuclear egress (Liu et al., 2014b). The HSV-1 proteins ICP27 and UL47 are both nucleocytoplasmic shuttling proteins and RNA binding proteins, and their binding with the poly(A)-binding protein PABP1 ultimately leads to Paip2 translocation and the nuclear accumulation of PABPC1. PAPBC1 plays an important role in effective translation initiation. Under normal circumstances, it binds to the poly(A) tail of mRNA in the cytoplasm and enhances the stability and translation efficiency of mRNA. Abnormal nuclear localization of PAPBC may affect the processing or export steps of viral or host mRNAs (Dobrikova et al., 2010). The lytic replication of herpesvirus is activated by the viral transcriptional activator VP16, which activates the immediate-early genes of the virus and initiates a complex cascade of gene expression (Johnson et al., 1999). Studies have shown that the PRV UL46 protein interacts with VP16 through the NLS to regulate the function of VP16 (Xu et al., 2020).

In β herpesvirus, the HCMV UL94 protein interacts with the HCMV pp28 protein through nuclear import sites to affect viral particle assembly (Liu et al., 2009). In addition, the HCMV IE2 protein is the main transactivator and is encoded by the HCMV gene. The HCMV UL84 protein interacts with the IE2 protein to interfere with IE2-mediated transactivation of early gene promoters. In HCMV UL84 NES non-shuttle mutant transfected cells, the IE2 protein presented an abnormal localization pattern in the nucleus and failed to be correctly assigned to the replication chamber. The nucleocytoplasmic shuttling of the UL84 protein may indirectly regulate IE2 translation and localization (Colletti et al., 2004). In γ herpesvirus, the expression of the EBV BFLF2 protein alone is limited to the nucleus, while the expression of the EBV BFRF1 protein alone is located in the cytoplasm and perinucleus, the two proteins are co-localized at the nuclear rim, and their interaction plays a key role in the viral envelopment (Lake and Hutt-Fletcher, 2004). In general, the interaction network among viral proteins is complex and extensive. The interactions between these proteins can influence their localization, expression, and function. Some shuttling proteins play a regulatory role by affecting both their own localization and that of other proteins.

## 5 Other functions

### 5.1 Herpesvirus shuttling proteins regulate apoptosis

Apoptosis is a type of programmed cell death associated with characteristic morphological and biochemical changes in cells. It plays a role in preventing virus transmission and spread in the early stages of viral infection while promoting virus replication and export in the later stages of viral infection (Zhou et al., 2017). In α herpesvirus, BHV-1 UL47 protein does not inhibit the phosphorylation of SMC1 in the cytoplasm, but it inhibits SMC1 phosphorylation in the nucleus, leading to disruption of the ATM/NBS1/SMC1 pathway and inhibition of DNA repair. SMC1 is a component of the DNA damage network. Studies have shown that blocking SMC1 phosphorylation reduces cell survival after DNA damage. In BHV-1 UL47 protein-transfected cells, DNA damage-induced apoptosis increased, so the BHV-1 UL47 protein inhibits SMC1 phosphorylation and DNA repair, demonstrating a potential role in regulating apoptosis (Kitagawa et al., 2004; Vasilenko et al., 2012; Afroz et al., 2018). BHV-1 VP22 is a tegument protein encoded by the UL49 gene. The mitochondria-targeting sequence at the C terminus of the VP22 protein is found within the VP22 NES (Zheng et al., 2005; Zhu et al., 2005). VP22 has been reported to induce apoptosis (Qiu et al., 2005), and mitochondria playing a central role in this process (Jeong and Seol, 2008). Therefore, it is reasonable to speculate that the VP22 protein is exported to the cytoplasm via the NES and subsequently enters the mitochondria to influence apoptosis. Furthermore, in γ herpesvirus, a portion of the KSHV genome specifically mediates the maintenance of the virus in the B-cell environment, LANA2 is a nuclear latent protein detected only in B cells infected with KSHV, which inhibits p53 tumor suppressor gene protein-dependent transcriptional transactivation and apoptosis, as well as PKR-dependent apoptosis (Chao et al., 2000; Rivas et al., 2001; Chipuk and Green, 2003). LANA2 NLS mutants resulting in increased cytoplasmic localization are unable to inhibit the apoptosis induced by p53 activation. Subsequently, it was found that LANA2 phosphorylation inhibits the function of NES and affects the ability to inhibit p53-dependent apoptosis. Indicating that the nuclear localization of the LANA2 protein is crucial for the inhibition of p53-induced apoptosis and LANA2's nucleocytoplasmic shuttling mediates its ectopic location to the nucleus to inhibit apoptosis and promote the proliferation of virions (Muñoz-Fontela et al., 2003, 2005). In general, some herpesvirus shuttling proteins affect node proteins in apoptosis-related pathways through nucleocytoplasmic shuttling to mediate apoptosis.

### 5.2 Herpesvirus shuttling proteins participate in immune escape

Herpesvirus infection is lifelong and highly host-specific, to resist virus invasion, hosts use pattern recognition receptors (PRRs), such as retinoic acid-inducible gene I (RIG-I) and melanoma differentiation-associated gene 5 (MDA5), to recruit a series of signal transduction molecules, such as stimulator of interferon genes (STING) and mitochondrial antiviral signaling protein (MAVS). These proteins transfer signals to downstream molecules in different signaling pathways, ultimately leading to the activation and translocation of multiple transcription factors, including NF-κB, IFN regulatory factor 3 (IRF3), and IFN regulatory factor 7 (IRF7), into the nucleus, inducing the expression of type I interferons (IFN-I) and proinflammatory cytokines to activate downstream JAK-STAT signaling pathways, which results in the expression of multiple interferon-stimulated genes (ISGs), ultimately resulting in antiviral immune response (Pestka et al., 2004; Stark and Darnell, 2012). Therefore, during latent infection, the herpesvirus gradually evolves various host immune evasion strategies to ensure its survival to evade the host immune response. Some herpesvirus shuttling proteins participate in the virus immune evasion process by inhibiting the production of IFN-I, blocking downstream IFN signaling, regulating specific ISGs, antagonizing the host antiviral innate immune response and achieving effective virus transmission and pathogenicity.

In α herpesvirus, the nuclear localization of the BHV-1 ICP27 protein is necessary to inhibit IFN-β promoter activity in transfected cells, and the BHV-1 ICP27 Δ N (Δ NLS+NoLS) mutation interferes with the ability to inhibit IFN-β1 and IFN-β3 promoter activity (da Silva et al., 2012). On the other hand, the BHV-1 ICP27 protein regulates 3′ mRNA processing, whether ICP27 affects immunity due to its potential interference with the 3′ processing of cellular factors necessary for IFN-β-dependent transcription remains to be investigated (Singh et al., 1996). Daxx is an important component of intrinsic cellular immunity, herpesviruses achieve replication and immune escape in host cells by regulating Daxx, and the HSV-1 ICP27 protein has been reported to ectopic Daxx from the nucleus to the cytoplasm, increasing the interaction between p65 NF-κB and Daxx, thereby inhibiting NF-κB activity and regulating innate immune processes (Schreiner and Wodrich, 2013; Kim et al., 2017). Transcription activator 1 (STAT1) is a potential cytoplasmic transcription factor that directly participates in the IFN-I-mediated signaling pathway. Studies have shown that NLS deficiency in the BHV-1 UL47 protein inhibits the nuclear accumulation of STAT1 after IFN-β stimulation and ultimately downregulates IFN-I signaling (Afroz et al., 2016). In addition, the HSV-1 ICP27 protein can also inhibit IFN-I signaling by inhibiting STAT1 phosphorylation and nuclear accumulation (Johnson et al., 2008). Further investigated how the cellular localization of ICP27 affects the inhibition of IFN expression by using ICP27 ΔNES or ICP27 ΔNLS mutant viruses and reported that full inhibition of the IFN response depends on the cytosolic position of ICP27 after shuttling (Christensen et al., 2016). PRV UL46 protein helps viruses evade host innate immunity by regulating STING function in the cytoplasm (Xu et al., 2020). The HSV-1 γ_1_34.5 protein interrupts the translocation of RIG-I from the cytoplasm to mitochondria and disrupts the translocation of STING from the endoplasmic reticulum to the Golgi apparatus, the γ_1_34.5 protein also inhibits IRF3 phosphorylation and nuclear translocation, resulting in downregulation of the IFN response (Verpooten et al., 2009; Pan et al., 2018; Liu et al., 2021).

In β herpesvirus, the HCMV UL94 protein inhibits the antiviral innate immune response by targeting STING. STING recruits TBK1 and IRF3, in which IRF3 is phosphorylated and activated by TBK1, resulting in the subsequent induction of IFN-I. The UL94 protein selectively impairs the recruitment of TBK1 to STING signalosomes, resulting in the inhibition of downstream signal transduction (Zou et al., 2020). In γ herpesvirus, the KSHV LANA2 protein mediates the nuclear translocation of NF-κB, affecting NF-κB activity (Seo et al., 2004). The KSHV ORF45 protein inhibits virus-induced IFN-I production by blocking its phosphorylation and nuclear translocation through interaction with IRF7 (Zhu et al., 2002). In summary, proteins shuttled by herpesvirus participate in immune escape by regulating the localization of signal transduction molecules and transcription factors in the cytoplasm or nucleus and ultimately affect IFN activity, demonstrating the clever use of the cellular regulatory mechanisms of viral proteins to antagonize innate immune responses.

### 5.3 Cytoskeleton rearrangement induced by herpesvirus shuttling proteins

The cytoskeleton is involved in maintaining cell integrity and structure and remodeling surface structure and movement, including actin filaments, microtubules, and intermediate filaments. Herpesvirus remodels actin during the process of host cell entry, assembly and release, and transmits between host cells by destroying the cytoskeleton (Miranda-Saksena et al., 2018). The US3 protein is a multifunctional protein that participates in the modification of the cytoskeleton and nuclear egress of the herpesvirus capsid during the viral replication cycle, directly interacting with actin regulatory mechanisms. In α herpesvirus, HSV-2 US3 is the determining factor involved in the reorganization of the actin cytoskeleton into filamentous processes (FPs), and the formation of FPs is related to an increase in the cell-to-cell spread of the virus infection. HSV-2 US3 inhibits the formation of FPs if it cannot be exported to the cytoplasm. Furthermore, NES mutations in HSV-2 US3 contribute to its kinase activity, which is required for HSV-2 US3-induced FP formation and disassembly of actin stress fibers, thus coupling the nucleocytoplasmic shuttling properties of US3 with functional play. In addition, the loss of the HSV-2 US3 protein disrupts nuclear egress (Finnen et al., 2010, 2011). In summary, the HSV-2 US3 protein triggers remodeling of the host cytoskeleton during the life cycle through nucleocytoplasmic shuttling to promote the effective entry and transmission of the virus into the host cell.

## 6 Conclusion and prospects

The replication process of herpesviruses includes adsorption, entry, uncoating, biosynthesis, virion assembly, maturation, and release. Herpesvirus shuttling proteins play important roles in regulating viral mRNA transport, new capsid assembly and primary envelopment. These viral proteins are transported to different compartments through nucleocytoplasmic shuttling and play a role in different stages of viral maturation, which is conducive to maximizing self-regulatory functions. Although there are few relevant reports, it is reasonable to speculate that nucleocytoplasmic shuttling is essential for some viral proteins to perform multiple functions. Some herpesvirus nucleocytoplasmic shuttling proteins hijack the host pathway to export viral mRNA, inhibit host mRNA export, promote protein expression necessary for further replication and assembly of new viral particles, and block the expression of host-encoded cellular defense protein mRNAs.

At present, many available information about the function of shuttling proteins in the lifecycle of herpesviruses is based on α herpesviruses, especially HSV-1. Although some of this information is about HCMV and KSHV, if more shuttling proteins from β and γ herpesviruses can be identified in future research, a greater understanding of how shuttling proteins coordinate the viral lifecycle and whether they have similar functions can be achieved. Most of the characterized herpesvirus shuttling proteins interact with host transport receptors through the NLS and NES, utilizing intracellular transport mechanisms to shuttle between the nucleus and cytoplasm, promote virus proliferation, and evade host antiviral responses. First, NLS mediate transport to the nucleus, where they participate in virus replication, gene expression regulation, and assembly; in the later stage, NLS rely mainly on the export of NES to the cytoplasm for further maturation. Dysfunction of the NLS or NES can block or damage the production of infectious viral particles, demonstrating the roles of the NLS and NES in the viral lifecycle. Nucleocytoplasmic transport involving NLS and NES (including the transport of viral capsids, viral genes, viral polymerases, and some transcriptional regulators) is an important factor in viral transmission and regulates host-virus interactions. In summary, as the shuttling proteins of herpesvirus play multiple functions throughout the virus lifecycle, further elucidation of their mechanisms provide important references for identifying therapeutic targets. Targeting the binding region between the shuttling proteins and their nuclear transport receptors to inhibit the expression of nucleocytoplasmic function has broad importance for the development of antiviral drugs.
